# Practical learning opportunities and changes in teachers’ self-efficacy beliefs: Does the development of bachelor student teachers’ competence differ before and during COVID-19?

**DOI:** 10.1007/s35834-022-00357-3

**Published:** 2022-09-07

**Authors:** Johannes König, Kristina Gerhard, Daniela J. Jäger-Biela

**Affiliations:** Empirical School Research, University of Cologne, Gronewaldstr. 2, 50931 Cologne, Germany

**Keywords:** COVID-19, Longitudinal study, Practical learning opportunities, Teacher education, Teacher self-efficacy beliefs

## Abstract

This article investigates changes in student teachers’ teacher self-efficacy beliefs (SEBs) as part of their competence development during their three-year bachelor’s program and how these changes can be explained through practical learning opportunities. Using two bachelor’s student teacher cohorts who studied during and prior to COVID-19, differences in the development of their competence are analyzed longitudinally over three time points. Unexpectedly, no increase in SEBs was observed; rather, a significant decline was identified, with little practical relevance for the cohort who had studied before and medium practical relevance for the cohort who studied during the pandemic. At the end of their bachelor’s program, students in the cohort who studied before the pandemic reported slightly higher levels of SEBs than those who had to study during the pandemic. A path model indicates that studying during the pandemic negatively impacted both practical learning opportunities and changes in student teachers’ SEBs, whereas practical learning opportunities positively impacted SEBs changes. The findings’ implications for practical learning opportunities in initial teacher education are discussed.

## Introduction

As in many other countries worldwide, universities and schools in Germany closed in 2020 and 2021, either temporarily or completely, as a result of the COVID-19 pandemic, and student teachers had to rely on digital learning opportunities. Moreover, student teachers in Germany have recently experienced severe cutbacks in terms of practical learning opportunities owing to school closures and the challenges that schools have had to confront in adapting to online teaching (König et al. 2020).

This paper examines how school closure constraints during the COVID-19 pandemic have affected student teachers’ competence development (Carrillo and Flores 2020). Student teachers’ teacher self-efficacy beliefs (SEBs) are examined as a core characteristic of their competence (Baumert and Kunter 2013) and as a research construct (e.g., Tschannen-Moran and Woolfolk Hoy 2001) that has been previously applied in numerous empirical studies as an outcome of student teachers’ practical learning. Practical learning opportunities are captured using students’ self-reports regarding 65 activities in four substantial dimensions: lesson planning, teaching, linking theories to situations, and reflecting on practice, thus covering the major demands placed on student teachers during higher education (Seifert and Schaper 2018). Using two different cohorts of bachelor students—one who graduated in 2018, the other in 2021—we investigate whether differences are evident in the development of student teachers’ competence in times of COVID-19 vs. before. Our research emphasizes the importance of supporting student teachers’ development of their professional competence during the COVID-19 pandemic to ensure that they have equal opportunities in becoming highly qualified teachers. Implications of the findings for designing practical learning opportunities in initial teacher education are discussed.

### Student teachers’ teacher self-efficacy beliefs

Perceived self-efficacy is defined as “people’s beliefs about their capabilities to exercise control over their own level of functioning and over events that affect their lives” (Bandura 1991, p. 257). As socio-cognitive theory regards these beliefs as domain- and situation-specific (Bandura 1997), SEBs have been related to the target group of teachers and the professional demands of teaching. Teachers’ SEBs concern teachers’ confidence in their abilities to successfully master classroom teaching and student learning (Klassen and Chiu 2010; Klassen et al. 2011; Tschannen-Moran et al. 1998; Tschannen-Moran and Woolfolk Hoy 2001). Research on teachers’ SEBs has substantially increased in recent decades, demonstrating its relevance for outcomes such as teachers’ instructional quality, students’ cognitive and motivational learning, and teachers’ well-being (e.g., Bach 2022; Klassen and Chiu 2010; Zee and Koomen 2016). Today, teacher self-efficacy is considered central to describing and analyzing teachers’ motivation and reflection on their abilities (e.g., Lauermann and ten Hagen 2021), and SEBs have been conceptualized as a key element of teacher competence (Baumert and Kunter 2013; Klassen et al. 2011; Miller et al. 2017).

Teachers’ SEBs may be shaped as early as during the initial stages of teacher education. Theoretically, this concerns “the personal transformation from student to teacher” that student teachers must confront as an overall developmental task (Klassen and Durksen 2014, p. 158; Ostermann 2014). Upon entry into initial teacher education, student teachers’ self-conception remains closely related to the role they held as students attending school, as their experience gained during that time is still paramount (Jordell 1987; Lortie 1975). Role-taking is an essential aspect of initial teacher education, which aims to foster student teachers’ professional development. This entails transforming the student’s perspective on teaching and school into a more professional teacher’s view (König et al. 2016). Consequently, over the course of initial teacher education programs, student teachers are required to repeatedly reflect on their self-conception as they progress toward professionalism (Arnold et al. 2011). For example, observing successful teacher–student interactions or early success during a practicum with positive feedback from mentors can support this transformation (Fackler and Malmberg 2016; Makrinus 2012; Morris et al. 2017). Because such experiences are considered “sources” of SEBs (Bandura 1997) as will be outlined in the following, they can enhance student teachers’ SEBs. Owing to teachers’ SEBs’ importance as a key characteristic of the professional teacher and an outcome of initial teacher education, they have been utilized as a measure in numerous evaluations of initial teacher education (e.g., Bach and Hagenauer 2021; Pendergast et al. 2011; Schüle et al. 2017).

### Change and development of teacher self-efficacy beliefs during teacher education

Initial teacher education programs are expected to support student teachers’ SEBs development to prepare them for professional teaching (e.g., Berg and Smith 2018; Tschannen-Moran and Woolfolk Hoy 2007; Woolfolk Hoy 2008; Yada et al. 2021). Numerous empirical studies have found that student teachers’ SEBs generally increase during teacher training (e.g., Garvis et al. 2012; Palmer 2006; Schulte et al. 2008; Seifert and Schaper 2018; Swan et al. 2011; Woolfolk Hoy and Burke Spero 2005). However, these findings are not homogeneous (e.g., Caires et al. 2012; Duffin et al. 2012; Schüle et al. 2017), and further empirical research is required to examine the change of student teachers’ SEBs during initial teacher education and to identify which factors best support its development (Duffin et al. 2012; van Dinther et al. 2011).

Teachers’ SEBs are considered subject to change during initial teacher education (Bandura 1997; Woolfolk Hoy and Burke Spero 2005); in particular, practical learning opportunities appear to be influential in the early increase of self-efficacy among student teachers (Eisfeld et al. 2020; Fives et al. 2007; Klassen and Durksen 2014; Schüle et al. 2017; Seifert and Schaper 2018). Practical learning opportunities usually relate to teaching practice (sometimes denoted as “field experience”, Tatto et al. 2012, p. 34, or “practice”, Ball and Forzani 2009). They are closer to vocational teaching than the academic coursework undertaken at university, thus approaching teacher professionalism proactively and responsibly (König et al. 2017a; Flores 2016; Sachs 2000). Teaching practice is essential for student teachers’ acquisition of expertise (Berliner 2004) and reflective practice (Schön 1983). Regarding the construct of teachers’ SEBs that specifically builds on experiences gained in the relevant domain (Bandura 1997), practical learning opportunities seem constitute an indispensable segment of teacher education programs with respect to the development of student teachers’ SEBs.

Practical learning opportunities are an essential component of many teacher education programs worldwide (Arnold et al. 2014; Flores et al. 2014). Student teachers are typically required to observe teaching and teach their first lessons as part of their training (Moulding et al. 2014). Corresponding experiences may be perceived as successful events by student teachers—denoted as “vicarious experiences” (teacher observation) and “mastery experiences” (teaching practice) (Bandura 1997; Klassen and Durksen 2014; Tschannen-Moran and Woolfolk Hoy 2007)—fostering their anticipation of success in future teaching situations. Such experiences serve as “sources” of SEBs (Bandura 1997). This learning and reflection process among student teachers may contribute to increased SEBs stability that extends into their early career and in-service teaching competence development.

Seifert and Schaper (2018) evaluated long-term practicum effects using a pre–post design at three German universities. Student teachers’ SEBs increased significantly (*d* = 0.50), and this change could be predicted by the amount of planning (β = 0.15, *p* < 0.05) and teaching activities (β = 0.17, *p* < 0.01) performed by the student teachers (Seifert and Schaper 2018, p. 212). However, other studies have yielded contradictory findings, identifying a decline that followed the increase (e.g., Woolfolk Hoy and Burke Spero 2005). Mastery experiences may be more successful than vicarious experiences in fostering SEBs (Pfitzner-Eden 2016a). Against this background, we conclude that the questions of how teacher SEBs change and develop during initial teacher education and how teacher education characteristics such as practical learning opportunities affect this development remain largely and merit further empirical investigation (Clark and Newberry 2019; Hascher and Hagenauer 2016).

### Initial teacher education during COVID-19 university and school closures

Aside from research desiderata relating specifically to the change and development of student teachers’ SEBs during initial teacher education, institutionalized processes of teaching and learning to teach have faced unprecedented challenges since the outbreak of the COVID-19 pandemic (Carrillo and Flores 2020), which gave rise to unfamiliar distance teaching and learning scenarios. In Germany, the need for teachers to adapt to online teaching was hindered by schools’ lack of equipment and resources, and teachers’ inadequate skills to cope with the digital transformation (König et al. 2020).

While many universities successfully transitioned to online teaching, student teachers’ practical learning opportunities were significantly affected by school closures. This impacted the design and implementation of teaching practice during initial teacher education, either eliminating it entirely or moving it online as an alternative. Whereas students might be provided with surrogate teaching observation (e.g., by using online videos), their learning to practice in real teaching context was most likely reduced to a larger extent (Wyss and Staub 2021). We therefore assume that the reduction of practical learning opportunities due to pandemic might generally be related to “mastery experiences” (teaching practice) rather than “vicarious experiences” (teacher observation) as important sources of SEBs (Bandura 1997).

The impact of partial school closures and reduced practical learning opportunities on student teachers’ SEBs development has emerged as a key research question that has practical relevance, as students generally evaluate practical learning opportunities as crucial for their professional growth (Arnold et al. 2011; Cohen et al. 2013; König and Rothland 2018).

## Research questions and hypotheses

Our study addresses the following research questions:

1. Do student teachers’ SEBs increase during their three-year bachelor program?
2. Do comparisons of student teachers before and during the COVID-19 pandemic reveal any differential SEB development?
3. Can the practical learning opportunities to which student teachers are exposed throughout their bachelor studies predict SEB changes?
4. Have the reduced practical learning opportunities during COVID-19 affected student teachers’ SEB development?

In line with existing research, we hypothesize a significant increase in student teachers’ SEBs over the course of their bachelor’s degrees (first hypothesis, H1). Focusing on three time points, each close to the end of the academic year, student teachers’ SEBs are expected to increase at the end of their three-year bachelor’s degree following a linear trend. Since the partial school closures caused by the COVID-19 pandemic resulted in the severe reduction of practical learning opportunities, a comparison of bachelor cohorts studying during and before COVID-19 will reveal a smaller increase in student teachers’ SEBs (H2).

The bachelor program under study comprises substantial practical learning opportunities, with the requirement that students complete two different practicum types. One practicum takes place outside school and relates to different forms of vocational explorations. The other practicum—more relevant to our study—takes place in school and serves to expose students to typical vocational teacher tasks and how teachers master these tasks in their daily work. Student teachers must serve in a school of the type they hope to teach in: for example, a primary school student teacher must be assigned to a primary school. The minimum practicum length is 25 days with 100 h of actual participation at school. The student teachers’ activities vary, but they typically observe lessons and teach under the supervision of an in-service teacher who serves as their mentor during the practicum. Student teachers may take on teaching responsibilities, though this is not a curricular requirement. However, they usually do (König et al. 2017b). Credits are given for a total workload of 180 h, including preparation and reflection as well as documenting practical learning opportunities. During the same semester, student teachers also attend a preparation methods course with a curriculum aligned with the following overarching topics: teaching, education, assessment, and professional development. Student teachers are required to document their practical learning experiences in portfolios and reflect on their practical learning as part of an evaluation meeting. In addition to these formal practical learning approaches, student teachers also enjoy “opportunities for enactment and experimentation (…) through the use of approximations of practice” (Grossman et al. 2009, p. 2076)—for example, role-playing in academic seminars.

The theoretical underpinnings and empirical evidence outlined here led us to hypothesize that change in student teacher SEBs can be significantly predicted based on the amount of practical learning opportunities (including approximations of practice) to which individual student teachers are exposed during their bachelor’s program (H3). Again, in a path modeling analysis, we assume an indirect effect on the cohort’s differential SEBs development owing to the reduction in opportunities for practical learning caused by the COVID-19 pandemic (H4).

## Methods

### Participants and procedures

**Fig. 1:**
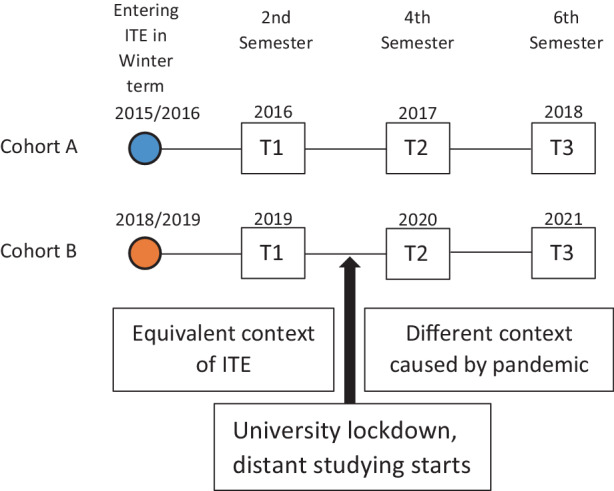
Data collection design. (*ITE* initial teacher education, *T* time point)

Data were collected as part of the Quality Assurance in the project Zukunftsstrategie Lehrer*innenbildung Köln (ZuS) [Strategy for the future of teacher education in Cologne], an empirical study on teacher preparation at the University of Cologne. This university is one of the largest higher education institutions providing teacher education in Germany. Two bachelor student teacher cohorts who had begun studying either in winter term 2015 (cohort A) or three years later in winter term 2019 (cohort B) were longitudinally sampled at three time points (T) each (T1: summer 2016/2019, T2: summer 2017/2020, T3: summer 2018/2021 for cohort A/B, see Fig. 1). Whereas cohort A represents the situation before the pandemic, cohort B was confronted with the pandemic in March 2020 prior to entering their fourth semester in the summer term, which at German universities usually begins in April. As their university did not return to partial opening until October 2021, the second half of cohort B’s bachelor degree differed significantly from that of cohort A, despite both cohorts having begun their initial teacher education programs under similar conditions. This allowed comparative analyses and the examination of differences in learning opportunities associated with the pandemic and possible differential effects of cohorts during their study as a result of these differences.

### Sample

A complete survey of student teachers in that specific semester was conducted at each time point, but the response rate was merely moderate (*Min*. 28.0%–*Max.* 43.7%, see Table 1). Student teachers were surveyed during compulsory lectures, thus preventing their individual self-selection during participation. However, only online surveys were allowed during the pandemic therefore decreasing cohort B response rate at T2 and T3, which must be regarded as a limitation for our comparison. Our sample presumably constitutes a positive selection of student teachers, since for example the population decreases over their three-year bachelor’s program (e.g., nearly every third cohort B student dropped out between T1 and T3, see Table 1). Panel mortality most likely occurred as well. Moreover, more female student teachers are represented in the surveys (around 80%) than could be expected from the population statistics (around 70%, see Table 1). Panel samples used in the analysis presented here include only 331 student teachers from both cohorts (cohort A/B: 210/111) who could be followed up at all three time points. The relatively small panel sample size reflects the practical difficulties we experienced in reaching student teachers and encouraging them to participate at all three time points.

**Table 1:** Population and sample statistics

Cohort	T1		T2		T3		Panel
	Population (female)	Sample (female)	Population (female)	Sample (female)	Population (female)	Sample (female)	
A	1,668 (68.6%)	729 (80.7%)	1,433 (69.8%)	605 (83.8%)	1,238 (69.1%)	428 (82.7%)	210
Response	–	43.7%	–	42.2%	–	34.6%	–
B	1,757 (69.9%)	668 (79.8%)	1,490 (70.0%)	429^a^ (82.1%)	1,205 (68.5%)	338^a^ (80.8%)	111
Response	–	38.0%	–	28.8%	–	28.0%	–

^a^Online survey during COVID-19 pandemic university closure

The student teachers surveyed represent all teacher education programs provided by the university. These programs prepare them for teaching in primary school (17%), lower secondary school (14%), grammar school (22%), vocational colleges (3%), or special needs education (42%). These proportions roughly correspond to the proportions in the population of T3 (cohort A/B: 11%/21%, 13%/17%, 23%/14%, 2%/6%, 51%/40%). In our sample, 89.4% of the students were female, 8.4% were male, 0.3% were diverse, and 1.9% provided other information about gender, with no significant difference between cohort panel samples (χ^2^ = 0.606, *df* = 3, *p* (two-tailed) = 0.895). The student teachers had a grade point average (*Abiturnote*) of *M* = 2.09 (*SD* = 0.53, *Min.* = 1.0, *Max.* = 3.6), with no significant mean difference between cohort panel samples (*F* (1,319) = 1.51, *p* = 0.22). At T1, student teachers were, on average, *M* = 20.7 years old (*SD* = 3.3, *Min.* = 18, *Max.* = 48) with no significant mean difference between cohort panel samples (*F* (1,317) = 0.41, *p* = 0.523).

### Instruments

**Fig. 2:**
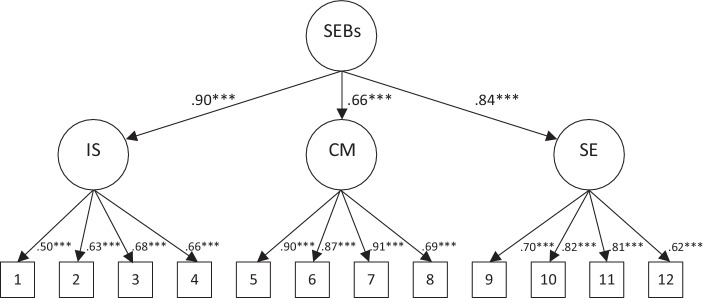
Findings from confirmatory factor analysis on teacher self-efficacy beliefs as a second-order factor overall score (standardized coefficients). (*SEBs* overall score teacher self-efficacy beliefs, *IS* teacher self-efficacy beliefs facet of instructional strategies, *CM* teacher self-efficacy beliefs facet of classroom management, *SE* teacher self-efficacy beliefs facet of student engagement. *Numbers* *1 to* *12* indicate the 12 items of Pfitzner-Eden’s (2016b) TSES instrument)

Student teachers’ SEBs were measured using Pfitzner-Eden’s (2016b) TSES instrument, consisting of 12 items and comprising three facets: instructional strategies, classroom management, and student engagement. Each facet was covered by four items (9-point Likert-scale from “not at all certain” to “absolutely certain”). However, previous studies (e.g., Berg and Smith 2014; Duffin et al. 2012) have called the differentiated three-factor structure of TSES into question for target groups like student teachers who have little teaching experience. As they suggest a general SEBs score as a better solution, we thus examined the factorial structure in our data. For this specific instrument analysis, the panel sample survey data from all time points and both cohorts were merged into a single file with a total of 993 cases (3 * 210 + 3 * 111). Using the software package *Mplus* (Muthén and Muthén 2017), a second-order confirmatory factor analysis (CFA) was performed using maximum likelihood estimation to simultaneously compare factor loadings of items and factor loadings of the overall score based on the three facets (Fig. 2). The model-based imputation option (full information maximum likelihood, FIML) was used to deal with missing data. The model fit was good (χ^2^ = 175.964, *df* = 51, *p* < 0.001; *CFI* = 0.970, *SRMR* = 0.038). As loadings for the second factor are relatively high (0.90/0.66/0.84) and following the principle of parsimony (e.g., Vandekerckhove et al. 2015), we conclude that an overall score of teachers’ SEBs may legitimately be used in our data. Subgroup analyses yielded insight into measurement invariance (configural invariance) of the second-order CFA (cohort A: χ^2^ = 103.109, *df* = 51, *p* < 0.001; *CFI* = 0.977, *SRMR* = 0.036; cohort B: χ^2^ = 133.273, *df* = 51, *p* < 0.001; *CFI* = 0.958, *SRMR* = 0.047).

Practical learning opportunities were surveyed at T3. We used an instrument that had already been applied in other studies of student teachers’ SEBs with large samples from various universities in Germany (e.g., Seifert and Schaper 2018). It comprises 65 items with which student teachers are required to report on their teaching practice activities. The items are classified into four dimensions (lesson planning, teaching, linking theories to situations, reflecting on practice), thus covering the major demands placed on student teachers during higher education. The instrument’s rationale was developed by König et al. (2014) with conceptual considerations drawn from theories regarding teachers’ professionalism and cognitive psychology as well as comprehensive analyses of teacher education curricular documents and expert reviews. The instrument has since been used in various empirical studies. For example, an in-depth factor analysis providing evidence for the four dimensions of the instrument was carried out by König et al. (2017b), using a sample of 1,347 bachelor student teachers in their fifth semester (i.e., a comparable target group to the sample of bachelor students in their sixth semester in the present study) from 18 universities or pedagogical colleges in Austria and Germany. Predictive validity could be provided for student teachers’ acquisition of general pedagogical knowledge (assessed via a standardized paper–pencil test at two time points) during initial teacher education in Austria and Germany (König and Klemenz 2015; König et al. 2017b).

The scale inventory introduces items using the initial question “During your in-school opportunities to learn, have you conducted the following activities?” and student teachers are asked to answer “yes” (coded as 1) or “no” (coded as 0), resulting in scale scores ranging from 0 to 1. Our data showed good scale reliability (Table 2). Using the four scales as manifest indicators that specify a latent variable, we performed a CFA, which indicated a good model fit (χ^2^ = 7.766, *df* = 2, *p* < 0.05; *CFI* = 0.988, *SRMR* = 0.019). Subgroup analysis provided insight into measurement invariance (cohort A: χ^2^ = 8.656, *df* = 2, *p* < 0.05; *CFI* = 0.980, *SRMR* = 0.024; cohort B: χ^2^ = 0.481, *df* = 2, *p* = 0.786; *CFI* = 1.000, *SRMR* = 0.009). This examination of the instrument’s factorial structure in the present analysis corresponds well with the psychometric properties reported in previous studies (König et al. 2014, 2017b; König and Klemenz 2015; Seifert and Schaper 2018).

**Table 2:** Item examples from the practical learning opportunities scales, number of items, and coefficients on scale reliability

Scale	Number of items	Item example	α	
			Cohort A	Cohort B
Lesson planning	12	I have formulated learning goals aligned to the curriculum	0.85	0.85
Teaching	31	I have checked attendance	0.93	0.93
Linking theories to situations	11	I have observed teaching methods that I have learned about during my university course	0.80	0.83
Reflecting on practice	11	I have drawn conclusions for future teaching	0.79	0.85

*α* Cronbach’s Alpha

### Data analysis

Repeated measure analyses of variance were conducted to test H1 and H2, and a path analysis is carried out to test H3 and H4.

## Findings

### Student teachers’ SEBs

**Fig. 3:**
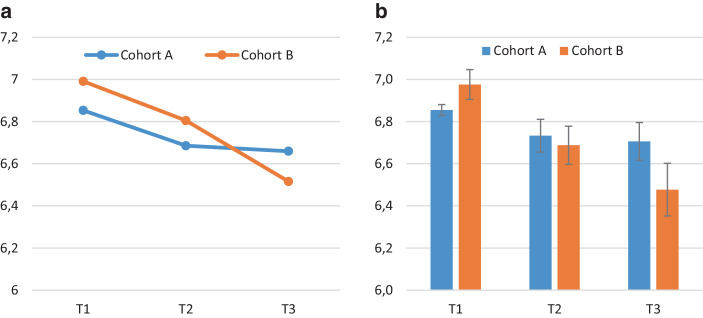
Student teachers’ SEBs per cohort (**a** means in the panel sample, **b** means and 95-% confidence interval in the full sample at each time point)

Table 3 and Fig. 3 present descriptive statistics for student teachers’ SEBs, indicating a decline between T1 and T2. While this decline persisted during the pandemic, student teachers who studied before pandemic showed no significant change in their SEBs between T2 and T3.

**Table 3:** Change in teachers’ SEBs (panel sample; means, *SD*, and *t*-test T1 vs. T2 vs. T3, T1 vs. T3 for each cohort A and B)

Cohort	T1	T2	T3	T1 vs. T2		T2 vs. T3		T1 vs. T3	
	*M* (*SD*)	*M* (*SD*)	*M* (*SD*)	*t*	*df*	*t*	*df*	*t*	*df*
A	6.85 (0.32)	6.70 (0.84)	6.64 (0.95)	3.03**	187	0.43	185	3.13**	198
B	6.99 (0.87)	6.81 (0.89)	6.52 (1.00)	2.16*	106	4.23***	108	5.76***	104

*p < 0.05, **p < 0.01, ***p < 0.001

To test H1, we conducted a repeated measure analysis of variance (ANOVA). No violation of sphericity was observed (Mauchly-*W* (2) = 0.985, *p* = 0.118). Time points had a significant influence on student teachers’ SEBs (*F* (2,576) = 17.77, *p* < 0.001, η_p_^2^ = 0.058). As the descriptive statistics in Fig. 3 show, this indicates the decline in SEBs among student teachers. Bonferroni pairwise mean difference comparisons show all mean differences as statistically significant. As a measure of effect size, *f* was computed (Cohen 1988), indicating a medium size effect (*f* = 0.25). As Table 3 demonstrates, the decrease in SEBs between T1 and T3 is significant, with small practical relevance for cohort A (*d* = 0.22 [0.08; 0.36]) but medium size in cohort B (*d* = 0.56 [0.36; 0.77]).

To test H2, we used this design and introduced cohort as a factor. This more comprehensive two-factor ANOVA design allows for the examination of differentiated evolution profiles between the cohorts by looking at the interaction effect, which is statistically significant (*F* (2,574) = 4.61, *p* < 0.05, η_p_^2^ = 0.016) with small practical relevance (*f* = 0.13).

The SEBs means were not significantly different at T3 (*F* (1,310) = 1.58, *p* = 0.21, η^2^ = 0.005); however, in the full sample (Fig. 3), they differ significantly with small practical relevance (*F* (1,707) = 9.01, *p* < 0.01, η^2^ = 0.013). At the end of their bachelor program, cohort B student teachers tend to indicate slightly lower levels of SEBs than cohort A student teachers. Moreover, means at each time point per cohort are almost identical in both the panel and the full sample, since the panel sample means fall well within the 95%-confidence interval of full sample means (Fig. 3). The only exception is cohort B’s SEBs mean at T2, which is slightly higher than expected within the full sample 95%-confidence interval. This underlines the positive selection of the panel sample but does not affect our interpretation of differential development between cohorts.

### Practical learning opportunities

**Fig. 4:**
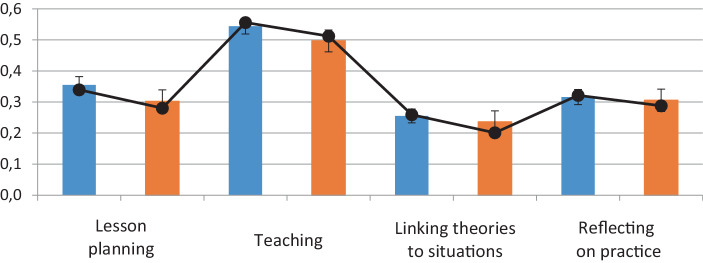
Means of practical learning opportunities per cohort (*bars*: full sample (cohort A: *blue color*, cohort B: *orange color*), *line*: panel sample)

Cohort B generally indicated fewer practical learning opportunities than cohort A. Comparison of the full samples reveals that cohort A’s means are significantly higher than those of cohort B (“lesson planning”: *t* (629) = 2.251, *p* = 0.025, “teaching”: *t* (629) = 2.149, *p* = 0.032). In the panel sample, however, significant mean differences could only be found for “linking theories to situations” (*F* (1,295) = 3.81, *p* = 0.05, η^2^ = 0.013) and mean differences at the 10% significance level for “planning” (*F* (1,297) = 3.13, *p* = 0.07, η^2^ = 0.010), each with cohort B students indicating that they had enjoyed fewer opportunities (Fig. 4).

### Path modeling

**Fig. 5:**
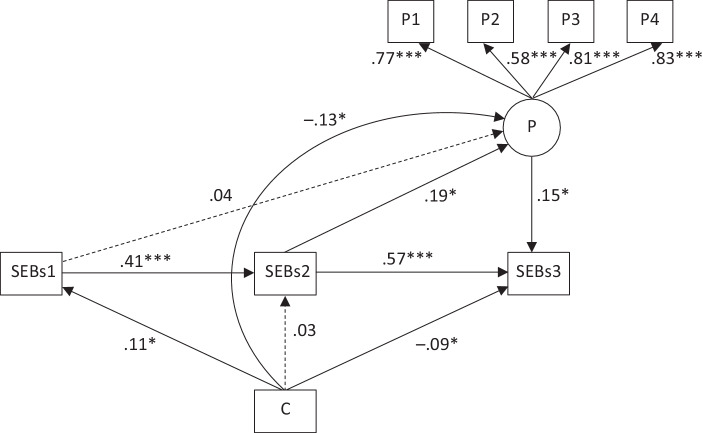
Findings from path modeling on teachers’ SEBs as an outcome (standardized coefficients). (*C* cohort (A = 0, B = 1), *P1* lesson planning, *P2* teaching, *P3* linking theories to situations, *P4* reflecting on practice, *SEBs1* self-efficacy beliefs time point 1, *SEBs2* self-efficacy beliefs time point 2, *SEBs3* self-efficacy beliefs time point 2)

To test H3 and H4, a path model was specified (Fig. 5). T3 student teachers’ SEBs was specified as a dependent variable and controlled for previous time points, allowing us to interpret the effects of other predictors as changes in SEBs over time. Having used SEBs as an overall measure, we refrained from specifying SEBs as a latent variable because of the relatively small size of the panel samples (cf. Bentler and Chou 1987). By contrast, practical learning opportunities were specified as latent variable and measured using the four scales (lesson planning, teaching, linking theories to situations, reflecting on practice) as indicators. Cohort assignment was included as a dichotomous variable, predicting SEBs at each time point as well as practical learning opportunities.

Again, we used the software package *Mplus* (Muthén and Muthén 2017), maximum likelihood estimation, and FIML to deal adequately with missing data. The overall model fit was good (χ^2^ = 45.731, *df* = 15, *p* < 0.001; *CFI* = 0.956, *SRMR* = 0.035). SEBs showed moderate stability over time (0.41, *p* < 0.001, between T1 and T2; 0.57, *p* < 0.001, between T2 and T3). Practical learning opportunities were shown to affect changes in SEBs (0.15, *p* < 0.05): the more practical learning opportunities the student teachers had, the stronger their agreement was on the SEBs scale. Cohort (1 = studying in times of COVID-19 vs. 0 = studying before) had direct negative effects on both practical learning opportunities (−0.13, *p* < 0.05) and changes in SEBs between T2 and T3 (−0.09, *p* < 0.05).

## Discussion

In this article, we analyzed changes in student teachers’ SEBs as part of their competence development during their three-year bachelor’s program and how these changes can be explained by the practical learning opportunities to which they have been exposed. Two different bachelor student teacher cohorts were included to determine whether the study conditions before and during COVID-19 exerted differential effects.

Against our expectations based on the literature survey, we observed no increase in the student teachers’ SEBs. Rather, we identified a significant decrease, with little practical relevance for cohort A and medium practical relevance for cohort B. At the end of their bachelor’s program, students who studied before the pandemic reported slightly higher SEBs than those who were obliged to study during the pandemic. Therefore, while H1 (increase in SEBs) was not confirmed, H2 (differential development) could be confirmed. Moreover, according to student teachers’ self-reports, studying during the pandemic was associated with fewer practical learning opportunities. For example, student teachers studying during the pandemic reported they had less opportunities to planning lessons (Fig. 4). A correspondent path model revealed that studying during the pandemic negatively affected both practical learning opportunities and the development of student teachers’ SEBs. We therefore concluded H3 (practical learning opportunities predicting the change in SEBs) and, owing to an additional direct cohort effect on practical learning opportunities, we also consider H4 to have been confirmed.

Regarding the unexpected finding in relation to H1, as Pfitzner-Eden (2016a) has shown, increased SEBs among student teachers is more likely to be related to a teaching practicum that is more conducive to students’ attainment of mastery (see also Seifert and Schaper 2018). Since the student teachers in our study were at least *formally* required to observe teaching rather than to teach by themselves, the vicarious experiences that they gained may have been the dominant source for SEBs development that their teacher education programs had offered them to date (Moulding et al. 2014). When giving priority to observing teaching during practicum, students may learn about the complexity of teaching as a profession for the first time (Bach 2022). Evaluating professional demands from the perspective now as beginning teacher, their reflections on teaching as a profession may negatively impact their SEBs (Pfitzner-Eden 2016a). However, the way student teachers cognitively process their vicarious or mastery experience plays an important role.

Therefore, comparison of different student teacher cohorts during a typical teaching practicum, such as the long-term practicum, before and during COVID-19 might have shown clearer effects on the change of SEBs. Besides, one should consider that student teachers already started out with relatively high levels of SEBs at the end of their second semester (i.e., T1 in our study design). With means near the value of 7, their agreement on the nine-point Likert-scale was, on average, very high, and the level that they showed at the end of their bachelor studies still indicated high average agreement. Consequently, their SEBs are strong. However, not necessarily the level per se but rather the differential development depending on learning opportunities was the specific focus of our study. Therefore, we were primarily interested in analyzing and reflecting on possible factors contributing to this development.

Regarding the student teachers’ practical activities, it is interesting to see that lesson planning was reduced during the pandemic (Fig. 4). As Seifert and Schaper (2018) demonstrated, the two dimensions of “lesson planning” and “teaching” significantly impact student teachers’ SEBs growth. Again, this reinforces the importance of vicarious and mastery experiences during practicum. Therefore, our findings demonstrate how specific reductions in practical learning opportunities have affected student teachers’ competence development. In particular, lesson planning is a challenging task for professional teachers and is a curricular aspect of several teacher preparation programs worldwide that should be systematically accounted for in teacher education (Rothland 2021). Both vicarious and mastery lesson planning experiences are regarded as key sources for SEBs development, gained through practical training from as early as bachelor’s teacher education programs. However, our analysis suggests that student teacher cohorts impacted by COVID-19 pandemic cutbacks have experienced deficits in key opportunities to develop their competence during their initial teacher education. The effects we detected are small, but the path analysis in particular facilitates a more comprehensive interpretation as it builds on multivariate data from two longitudinal samples. We conclude that the significant effects we identified are clearly of practical relevance and should be given careful attention in educational policy. In any case, the return to unrestricted practical learning opportunities for student teachers is crucial and should be assured once the pandemic has been brought under control.

## Limitations

Our study has several limitations that must be acknowledged. First, constraints in recruiting the samples make it difficult to definitively judge the quality of our data. Aside from population dropout, positive self-selection likely occurred when the student teachers were invited to participate in the online surveys during university closures. Cohort B students might thus be regarded as having been more motivated and more committed to participating. Whether this bias caused underestimation of differences between the two cohorts, including the differential decline in SEBs during the pandemic, remains an open question, and representative survey data might have yielded stronger evidence for a COVID-19 pandemic cohort effect. Second, changes in SEBs might occur further into initial teacher education—that is, among master’s student teachers or during induction (e.g., Pfitzner-Eden 2016a; Seifert and Schaper 2018). Therefore, it is not possible to generalize our findings to other segments of initial teacher education. Third, information on practical learning opportunities was captured exclusively using students’ self-reports, prompting the question of what differences were present in terms of institutional provision for the two respective cohorts. Finally, we included student teachers from a single large university in Germany only, making it difficult to generalize our findings across higher education institutions that implemented different closure strategies during the COVID-19 pandemic.

## References

[CR1] Arnold, K.-H., Hascher, T., Messner, R., Niggli, A., Patry, J.-L., & Rahm, S. (2011). *Empowerment durch Schulpraktika: Perspektiven wechseln in der Lehrerbildung*. Bad Heilbrunn: Klinkhardt.

[CR2] Arnold, K.-H., Gröschner, A., & Hascher, T. (Eds.). (2014). *Schulpraktika in der Lehrerbildung: Theoretische Grundlagen, Konzeptionen, Prozesse und Effekte*. Münster: Waxmann.

[CR3] Bach, A. (2022). *Selbstwirksamkeit im Lehrberuf: Entstehung und Veränderung sowie Effekte auf Gesundheit und Unterricht*. Münster: Waxmann.

[CR4] Bach, A., & Hagenauer, G. (2021). Emotionen im Praktikum als Quelle der Selbstwirksamkeitsüberzeugungen von Lehramtsstudierenden. In M. Carmignola & D. Martinek (Eds.), *Persönlichkeit – Motivation – Entwicklung: Festschrift für Franz Hofmann*. Verlag Dr. Kovac.

[CR5] Ball, D. L., & Forzani, F. M. (2009). The work of teaching and the challenge for teacher education. *Journal of Teacher Education*, *60*(5), 497–511.

[CR9] Bandura, A. (1991). Social cognitive theory of self-regulation. *Organizational Behavior and Human Decision Processes*, *50*(2), 248–287.

[CR10] Bandura, A. (1997). *Self-efficacy: The exercise of control*. New York: Freeman.

[CR11] Baumert, J., & Kunter, M. (2013). The COACTIV model of teachers’ professional competence. In M. Kunter, et al. (Ed.), *Cognitive activation in the mathematics classroom and professional competence of teachers* (pp. 25–48). Boston: Springer.

[CR6] Bentler, P. M., & Chou, C. P. (1987). Practical issues in structural modeling. *Sociological Methods & Research*, *16*, 78–117.

[CR69] Berg, D. A., & Smith, L. F. (2014). Pre-service teachers’ efficacy beliefs and concerns in Malaysia, England and New Zealand. *Issues in Educational Research*, *24*(1), 21–40.

[CR7] Berg, D. A., & Smith, L. F. (2018). The effect of school-based experience on preservice teachers’ self-efficacy beliefs. *Issues in Educational Research*, *28*(3), 530–544.

[CR8] Berliner, D. C. (2004). Describing the behavior and documenting the accomplishments of expert teachers. *Bulletin of Science, Technology, and Society*, *24*, 200–212.

[CR12] Caires, S., Almeida, L., & Vieira, D. (2012). Becoming a teacher: Student teachers’ experiences and perceptions about teaching practice. *European Journal of Teacher Education*, *35*(2), 163–178.

[CR13] Carrillo, C., & Flores, M. A. (2020). COVID-19 and teacher education: A literature review of online teaching and learning practices. *European Journal of Teacher Education*, *43*(4), 466–487.

[CR14] Clark, S., & Newberry, M. (2019). Are we building preservice teacher self-efficacy? A largescale study examining teacher education experiences. *Asia-Pacific Journal of Teacher Education*, *47*(1), 32–47.

[CR16] Cohen, J. (1988). *Statistical power analysis for the social sciences* (2nd edn.). Hillsdale: Erlbaum.

[CR15] Cohen, E., Hoz, R., & Kaplan, H. (2013). The practicum in preservice teacher education: A review of empirical studies. *Teaching Education*, *24*(4), 34–380.

[CR17] Duffin, L. C., French, B. F., & Patrick, H. (2012). The teachers’ sense of efficacy scale: Confirming the factor structure with beginning pre-service teachers. *Teaching and Teacher Education*, *28*(6), 827–834.

[CR18] Eisfeld, M., Raufelder, D., & Hoferichter, F. (2020). Wie sich Lehramtsstudierende in der Entwicklung ihres berufsbezogenen Selbstkonzepts und ihrer Selbstwirksamkeitserwartung in neuen reflexiven Praxisformaten von Studierenden in herkömmlichen Schulpraktika unterscheiden: Empirische Ergebnisse einer landesweiten Studie in Mecklenburg-Vorpommern. *Herausforderung Lehrer* innenbildung-Zeitschrift zur Konzeption, Gestaltung und Diskussion*, *3*(1), 48–66.

[CR19] Fackler, S., & Malmberg, L. E. (2016). Teachers’ self-efficacy in 14 OECD countries: Teacher, student group, school and leadership effects. *Teaching and teacher education*, *56*, 185–195.

[CR20] Fives, H., Hamman, D., & Olivarez, A. (2007). Does burnout begin with student-teaching? Analyzing efficacy, burnout, and support during the student-teaching semester. *Teaching and Teacher Education*, *23*(6), 916–934.

[CR21] Flores, M. A. (2016). Teacher education curriculum. In J. Loughran & M. L. Hamilton (Eds.), *International Handbook of Teacher Education* (pp. 187–230). Dordrecht: Springer.

[CR22] Flores, M. A., Santos, P., Fernandes, S., & Pereira, D. (2014). Pre-service teachers’ views of their training: Key issues to sustain quality teacher education. *Journal of Teacher Education for Sustainability*, *16*(2), 39–53.

[CR23] Garvis, S., Pendergast, D., & Keogh, J. (2012). Changes in teacher self-efficacy in the first year of primary school teacher education study. *The Journal of the World Universities Forum*, *5*(1), 87–95.

[CR24] Grossman, P., Compton, C., Igra, D., Ronfeldt, M., Shanahan, M. E., & Williamson, P. W. (2009). Teaching practice: A cross-professional perspective. *Teachers College Record*, *111*(9), 2055–2100.

[CR25] Hascher, T., & Hagenauer, G. (2016). Openness to theory and its importance for student teachers’ self-efficacy, emotions and classroom behaviour in the practicum. *International Journal of Educational Research*, *77*, 15–25.

[CR26] Jordell, K. Ø. (1987). Structural and personal influences in the socialization of beginning teachers. *Teaching and Teacher Education*, *3*(3), 165–177.

[CR27] Klassen, R. M., & Chiu, M. M. (2010). Effects on teachers’ self-efficacy and job satisfaction: Teacher gender, years of experience, and job stress. *Journal of Educational Psychology*, *102*(3), 741.

[CR28] Klassen, R. M., & Durksen, T. L. (2014). Weekly self-efficacy and work stress during the teaching practicum: a mixed methods study. *Learning and Instruction*, *33*, 158–169.

[CR29] Klassen, R. M., Tze, V., Betts, S. M., & Gordon, K. A. (2011). Teacher efficacy research 1998–2009: Signs of progress or unfulfilled promise? *Educational Psychology Review*, *23*(1), 21–43.

[CR32] König, J., & Klemenz, S. (2015). Der Erwerb von pädagogischem Wissen bei angehenden Lehrkräften in unterschiedlichen Ausbildungskontexten: Zur Wirksamkeit der Lehrerausbildung in Deutschland und Österreich. *Zeitschrift für Erziehungswissenschaft*, *18*(2), 247–277.

[CR35] König, J., & Rothland, M. (2018). Das Praxissemester in der Lehrerbildung: Stand der Forschung und zentrale Ergebnisse des Projekts *Learning to Practice*. In J. König, M. Rothland & N. Schaper (Eds.), *Learning to Practice, Learning to Reflect? Ergebnisse aus der Längsschnittstudie LtP zur Nutzung und Wirkung des Praxissemesters in der Lehrerbildung* (pp. 1–62). Wiesbaden: Springer VS.

[CR36] König, J., Tachtsoglou, S., Darge, K., & Lünnemann, M. (2014). Zur Nutzung von Praxis: Modellierung und Validierung lernprozessbezogener Tätigkeiten von angehenden Lehrkräften im Rahmen ihrer schulpraktischen Ausbildung. *Zeitschrift für Bildungsforschung*, *4*(1), 3–22.

[CR34] König, J., Rothland, M., Tachtsoglou, S., & Klemenz, S. (2016). Comparing the change of teaching motivations among preservice teachers in Austria, Germany, and Switzerland: Do in-school learning opportunities matter? *International Journal of Higher Education*, *5*(3), 91–103.

[CR30] König, J., Bremerich-Vos, A., Buchholtz, C., Lammerding, S., Strauß, S., Fladung, I., & Schleiffer, C. (2017a). Modelling and validating the learning opportunities of preservice language teachers: On the key components of the curriculum for teacher education. *European Journal of Teacher Education*, *40*(3), 394–412.

[CR33] König, J., Ligtvoet, R., Klemenz, S., & Rothland, M. (2017b). Effects of opportunities to learn in teacher preparation on future teachers’ general pedagogical knowledge: Analyzing program characteristics and outcomes. *Studies in Educational Evaluation*, *53*, 122–133.

[CR31] König, J., Jäger-Biela, D., & Glutsch, N. (2020). Adapting to online teaching during COVID-19 school closure: Teacher education and teacher competence effects among early career teachers in Germany. *European Journal of Teacher Education*, *43*(4), 608–622.

[CR37] Lauermann, F., & ten Hagen, I. (2021). Do teachers’ perceived teaching competence and self-efficacy affect students’ academic outcomes? A closer look at student-reported classroom processes and outcomes. *Educational Psychologist*, *56*(4), 265–282.

[CR39] Lortie, D. (1975). *Schoolteacher: A sociological study*. Chicago: University of Chicago Press.

[CR40] Makrinus, L. (2012). *Der Wunsch nach mehr Praxis: Zur Bedeutung von Praxisphasen im Lehramtsstudium*. Springer.

[CR41] Miller, A. D., Ramirez, E. M., & Murdock, T. B. (2017). The influence of teachers’ self-efficacy on perceptions: Perceived teacher competence and respect and student effort and achievement. *Teaching and Teacher Education*, *64*, 260–269.

[CR42] Morris, D. B., Usher, E. L., & Chen, J. A. (2017). Reconceptualizing the sources of teaching self-efficacy: A critical review of emerging literature. *Educational Psychology Review*, *29*(4), 795–833.

[CR43] Moulding, L. R., Stewart, P. W., & Dunmeyer, M. L. (2014). Pre-service teachers’ sense of efficacy: relationship to academic ability, student teaching placement characteristics, and mentor support. *Teaching and Teacher Education*, *41*, 60–66.

[CR44] Muthén, L. K., & Muthén, B. O. (2017). *Mplus user’s guide* (8th edn.). Los Angeles: Muthén & Muthén.

[CR45] Ostermann, E. (2014). *LehrerIn werden im Spannungsfeld subjektiver Erwartungen und objektiver Ausbildungsanforderungen: professionsspezifische Entwicklungsaufgaben für Lehramtsstudierende*. Bad Heilbrunn: Julius Klinkhardt.

[CR46] Palmer, D. (2006). Durability of changes in self-efficacy of preservice primary teachers. *International Journal of Science Education*, *28*(6), 655–671.

[CR47] Pendergast, D., Garvis, S., & Keogh, J. (2011). Pre-service student-teacher self-efficacy beliefs: an insight into the making of teachers. *Australian Journal of Teacher Education*, *36*(12), 46–57.

[CR49] Pfitzner-Eden, F. (2016a). Why do I feel more confident? Bandura’s sources predict preservice teachers’ latent changes in teacher self-efficacy. *Frontiers in Psychology*, *7*, 1–16. 27807422 10.3389/fpsyg.2016.01486 PMC5070217

[CR50] Pfitzner-Eden, F. (2016b). STSE. Scale for Teacher Self-Efficacy – deutsche adaptierte Fassung. In ZPID (Ed.), *Elektronisches Testarchiv*. Trier: ZPID.

[CR51] Rothland, M. (2021). Notes on modelling and operationalising (general didactic) lesson planning competence. *Unterrichtswissenschaft*. 10.1007/s42010-021-00111-0.

[CR52] Sachs, J. (2000). The activist professional. *Journal of Educational Change*, *1*(1), 77–95.

[CR55] Schön, D. A. (1983). *The reflective practitioner: How professionals think in action*. New York: Basic Books.

[CR53] Schüle, C., Besa, K.-S., Schriek, J., & Arnold, K.-H. (2017). Die Veränderung der Lehrerselbstwirksamkeitsüberzeugung in Schulpraktika. *Zeitschrift für Bildungsforschung*, *7*(1), 23–40.

[CR54] Schulte, K., Bögeholz, S., & Watermann, R. (2008). Selbstwirksamkeitserwartungen und pädagogisches Professionswissen im Verlauf des Lehramtsstudiums. *Zeitschrift für Erziehungswissenschaft*, *11*(2), 268–287.

[CR56] Seifert, A., & Schaper, N. (2018). Die Veränderung von Selbstwirksamkeitserwartungen und der Berufswahlsicherheit im Praxissemester. In J. König, M. Rothland & N. Schaper (Eds.), *Learning to Practice, Learning to Reflect?* (pp. 195–222). Wiesbaden: Springer VS.

[CR57] Swan, B., Wolf, K., & Cano, J. (2011). Changes in teacher self-efficacy from the student teaching experience through the third year of teaching. *Journal of Agricultural Education*, *52*, 128–139.

[CR58] Tatto, M. T., Schwille, J., Senk, S., Ingvarson, L., Rowley, G., Peck, R., Bankov, K., Rodriguez, M., & Reckase, M. (2012). *Policy, practice, and readiness to teach primary and secondary mathematics in 17 countries. Findings from the IEA teacher education and development study in mathematics (TEDS-M)*. Amsterdam: IEA.

[CR60] Tschannen-Moran, M., & Woolfolk Hoy, A. (2001). Teacher efficacy: capturing an elusive construct. *Teaching and Teacher Education*, *17*(7), 783–805.

[CR61] Tschannen-Moran, M., & Woolfolk Hoy, A. (2007). The differential antecedents of self-efficacy beliefs of novice and experienced teachers. *Teaching and Teacher Education*, *23*(6), 944–956.

[CR59] Tschannen-Moran, M., Woolfolk Hoy, A., & Hoy, W. K. (1998). Teacher efficacy: its meaning and measure. *Review of Educational Research*, *68*(2), 202–248.

[CR63] van Dinther, M., Dochy, F., & Segers, M. (2011). Factors affecting students’ self-efficacy in higher education. *Educational Research Review*, *6*(2), 95–108.

[CR62] Vandekerckhove, J., Matzke, D., & Wagenmakers, E. J. (2015). Model comparison and the principle of parsimony. In J. R. Busemeyer, Z. Wang, J. T. Townsend & A. Eidels (Eds.), *The Oxford handbook of computational and mathematical psychology* (pp. 300–319). Oxford: Oxford University Press.

[CR64] Woolfolk Hoy, A. (2008). What motivates teachers? Important work on a complex question. *Learning and Instruction*, *18*(5), 492–498.

[CR65] Woolfolk Hoy, A., & Burke Spero, R. (2005). Changes in teacher efficacy during the early years of teaching: A comparison of four measures. *Teaching and Teacher Education*, *21*(4), 343–356.

[CR66] Wyss, C., & Staub, S. (2021). Berufspraktische Lehrpersonenbildung während der Covid-19-Pandemie: Herausforderungen, neue Lernfelder und Entwicklungspotenzial. *Beiträge zur Lehrerinnen- und Lehrerbildung*, *39*(3), 320–331.

[CR67] Yada, A., Björn, P. M., Savolainen, P., Kyttälä, M., Aro, M., & Savolainen, H. (2021). Pre-service teachers’ self-efficacy in implementing inclusive practices and resilience in Finland. *Teaching and Teacher Education*, *105*, 103398.

[CR68] Zee, M., & Koomen, H. M. (2016). Teacher self-efficacy and its effects on classroom processes, student academic adjustment, and teacher well-being: a synthesis of 40 years of research. *Review of Educational Research*, *86*(4), 981–1015.

